# How ubiquitous is the direct-gaze advantage? Evidence for an averted-gaze advantage in a gaze-discrimination task

**DOI:** 10.3758/s13414-020-02147-3

**Published:** 2020-11-01

**Authors:** Eva Riechelmann, Matthias Gamer, Anne Böckler, Lynn Huestegge

**Affiliations:** 1 Department of Psychology, Würzburg University, Röntgenring 11, 97070 Würzburg, Germany; 2 Department of Psychology, Leibniz University Hannover, Hannover, Germany

**Keywords:** Social cognition, Gaze processing, Averted gaze, Direct gaze, Gaze discrimination

## Abstract

Human eye gaze conveys an enormous amount of socially relevant information, and the rapid assessment of gaze direction is of particular relevance in order to adapt behavior accordingly. Specifically, previous research demonstrated evidence for an advantage of processing direct (vs. averted) gaze. The present study examined discrimination performance for gaze direction (direct vs. averted) under controlled presentation conditions: Using a backward-masking gaze-discrimination task, photographs of faces with direct and averted gaze were briefly presented, followed by a mask stimulus. Additionally, effects of facial context on gaze discrimination were assessed by either presenting gaze direction in isolation (i.e., by only showing the eye region) or in the context of an upright or inverted face. Across three experiments, we consistently observed a *facial context effect* with highest discrimination performance for faces presented in upright position, lower performance for inverted faces, and lowest performance for eyes presented in isolation. Additionally, averted gaze was generally responded to faster and with higher accuracy than direct gaze, indicating an *averted-gaze advantage*. Overall, the results suggest that direct gaze is not generally associated with processing advantages, thereby highlighting the important role of presentation conditions and task demands in gaze perception.

## Introduction

The direction of human eye gaze conveys a significant amount of relevant social information, especially with respect to attention and intention, and serves as a reliable source of non-verbal information (Argyle & Cook, 1976; Emery, 2000; Frischen, Bayliss, & Tipper, 2007; Kendon & Cook, 1969; Kleinke, 1986; Schilbach, 2015). As such, gaze direction of others can point the gaze recipient towards sources of potential interest, or alert the gaze recipient about potential dangers, like the occurrence of an enemy. Therefore, it is obvious that quickly determining other persons’ gaze directions can be of high relevance in social situations and comes with an adaptive advantage given the potentially disastrous consequences of non-detection. Many studies have examined face- and gaze-processing mechanisms, thereby touching upon the topic of gaze discrimination. However, fewer studies focused on the systematic investigation of humans’ ability to discriminate gaze direction. The present study reports a series of three experiments dedicated to fill this research gap. Given that gaze discrimination is of special importance in situations of danger or threat, where “every second counts” and viewing conditions might be suboptimal, we put emphasis on the investigation of gaze discrimination ability when the perception of gaze direction is limited to a very short time window, that is, under brief and masked presentation conditions. Such conditions might be considered paradigmatic for real-life instances where gaze perception can be difficult due to situational constraints (e.g., time pressure, spatial distance, low lighting, etc.). Further, the present experiments are dedicated to investigating effects of facial context information on gaze discrimination ability.

### Social functions of gaze

In social interactions, gaze cues serve several purposes, for example providing information, guiding social interaction, fostering communication, expressing intimacy, and supporting goal-setting (Csibra & Gergely, 2009; Kleinke, 1986; Patterson, 1982; Richardson & Dale, 2005; Riechelmann, Raettig, Böckler, & Huestegge, 2019). Consequently, it comes as no surprise that humans are highly sensitive to gaze cues. Researchers have provided striking evidence that the perception of both direct and averted gaze direction affects social cognition. Perceiving *averted* gaze is assumed to trigger a quasi-reflexive shift of attention into the respective gaze direction (Driver et al., 1999; Friesen & Kingstone, 1998; Mansfield, Farroni, & Johnson, 2003; see Frischen et al., 2007, for a review on gaze cueing), whereas seeing a face with *direct* gaze can capture the attention of the observer. More specifically, it has been shown that presenting faces that are directly looking at the observer (vs. faces that are looking away) modulate cognitive processes, related to, for example, action control (Sato & Itakura, 2013; but see Riechelmann, Weller, Huestegge, Böckler, & Pfister, 2019) or to face perception in terms of faster recognition and better memorization, thereby establishing a *direct-gaze advantage* (Böckler, van der Wel, & Welsh, 2014; Boyer & Wang, 2018; Hood, Macrae, Cole-Davies, & Dias, 2003; Macrae, Hood, Milne, Rowe, & Mason, 2002; Senju & Hasegawa, 2005).

Despite the powerful function of *averted* gaze to automatically direct visual attention of the perceiver toward the direction of gaze, a strong line of research has emphasized the crucial role of (perceived) *direct* gaze as a socially relevant signal that indicates potential social interaction and therefore receives privileged visual attention (Chen & Yeh, 2012; von Grünau & Anston, 1995; Palanica & Itier, 2011, 2012; Senju, Hasegawa, & Tojo, 2005; Senju & Johnson, 2009; Stein, Senju, Peelen, & Sterzer, 2011). Findings from cognitive neuroscience that found modulations of brain activity by the perceived gaze direction in regions that are known to be involved in gaze processing (e.g., superior temporal sulcus or amygdala) support the claim that the perceptual system is specialized for the detection of and the attention to direct gaze (e.g., Hoffman & Haxby, 2000; Kawashima et al., 1999; but see Itier & Batty, 2009, for a review). von Grünau and Anston (1995) applied a visual search paradigm to empirically demonstrate this saliency of direct gaze – the “stare-in-the-crowd” effect. In their experiments, participants were presented with displays that showed eye stimuli in various gaze directions (straight vs. left- and rightward gaze). Participants had to indicate via keypress the presence or absence of a target (e.g., straight gaze) among distractors (e.g., left- and rightward gaze). When the task was to detect straight gaze among distractors of left- and rightward gaze, participants were faster compared to the detection of averted gaze targets among their distractors. A modified replication of the von Grünau and Anston (1995) study replicated the original findings (Senju et al., 2005). By including laterally averted faces as stimuli (instead of an isolated pairs of eyes) that looked either directly at the participant or away from the participant (to the left or right), the study of Senju et al. (2005) ruled out low-level perceptual characteristics as confounding factors for the original results, such as symmetry of straight gaze. On top of that, several studies provided evidence for an enhanced unconscious processing of direct (vs. averted) gaze on both behavioral (Chen & Yeh, 2012; Madipakkam, Rothkirch, Guggenmos, Heinz, & Sterzer, 2015; Stein et al., 2011) and neural (Yokoyama, Noguchi, & Kita, 2013) levels. Evidence for a privileged processing of direct (vs. averted) gaze was obtained for the presentation of both whole faces and isolated eye regions, irrespective of the level of processing (conscious vs. unconscious) (Chen & Yeh, 2012; Senju et al., 2005). This sensitivity to gaze direction and a privileged processing of direct gaze has been shown to arise very early in human development. Studies with infants observed that even neonates preferred to look at faces with direct gaze over faces with averted gaze or closed eyes (Batki, Baron-Cohen, Wheelwright, Connellan, & Ahluwalia, 2000; Farroni, Csibra, Simion, & Johnson, 2002; Vecera & Johnson, 1995). In sum, the abovementioned studies provide cumulative evidence for a general direct-gaze advantage and emphasize the social relevance for humans to be able to detect as quickly and accurately as possible if somebody is looking at them.

### Cues to gaze discrimination

Based on the considerations and findings reviewed above, it appears vital to ask what kind of cues humans use to discriminate gaze directions. In response to this issue, it has been suggested that the visual system integrates several factors to derive gaze direction judgments, depending on specific task requirements, thus establishing a multiple-cue account to gaze perception (Anderson, Risko, & Kingstone, 2016; Gamer & Hecht, 2007; Olk, Symons, & Kingstone, 2008). These include cues from the eye region, for instance, geometrical configuration of the eyes (Anstis, Mayhew, & Morley, 1969), luminance distribution (Anderson et al., 2016; Ando, 2002, 2004; Olk et al., 2008), or motion signals (Anderson et al., 2016), as well as cues outside of the eye region, for instance, head orientation (Balsdon & Clifford, 2017; Gamer & Hecht, 2007; Langton, 2000; Langton, Watt, & Bruce, 2000; Ricciardelli & Driver, 2008) or torso orientation (Seyama & Nagayama, 2005). The use of geometrical cues to detect gaze direction implies that gaze direction is derived from the relative position of the iris within the surrounding sclera and eyelids (Anstis et al., 1969). The human eye is characterized by a particularly salient contrast between the dark iris and the bright sclera, inducing a specific luminance distribution across the eye (Emery, 2000). When changing this luminance distribution, gaze discrimination ability is severely disrupted (Ando, 2002, 2004; Ricciardelli, Bayliss, & Driver, 2000; Sinha, 2000). As a consequence, it has been suggested that the visual system follows the rule that the darker region of the eye is a key feature for gaze direction estimation.

### The present study

Given that humans are highly sensitive to gaze cues, the present series of experiments was designed to examine the ability of humans to discriminate direct from averted gaze direction in varying face contexts under controlled presentation conditions. In our experimental setup, a gaze stimulus was presented for 35 ms, and masked afterwards in order to control the time available to process the stimulus (see Langton, Honeyman, & Tessler, 2004, for a similar gaze-discrimination task). As we were mainly interested in the processing of briefly presented face stimuli, the duration of the gaze stimulus was set such that gaze processing was at the lower boarder of conscious perception (cf. Kiss & Eimer, 2008; Pegna, Landis, & Khateb, 2008). Thus, the very short image duration used in the current study is likely to introduce a certain degree of uncertainty about the sensory input. Given that perception studies have shown the potential impact of facial context on gaze processing (see below), we decided to examine the effect of facial context on gaze discrimination accuracy by presenting gaze direction either in the context of an upright face, an inverted face, or in isolation (i.e., by only displaying the eye region). Presenting faces upside down is known to disrupt holistic face processing (Bruce & Langton, 1994; Yin, 1969) and to impair gaze discrimination accuracy (Jenkins & Langton, 2003; Vecera & Johnson, 1995). Participants were asked to indicate as quickly and accurately as possible the gaze direction of the presented stimulus via keypress. The present approach is methodically different from other studies on the detection of perceived direct (vs. averted) gaze where the target is not briefly, but continuously presented, for example among distractors during a visual search task (von Grünau & Anston, 1995; Senju et al., 2005). To the best of our knowledge, it is still an open issue how discrimination performance for categorizing gaze directions would differ depending on task type (visual search/gaze detection as investigated previously vs. gaze discrimination as studied in the present experiments).

We addressed our research questions by means of five dependent measures. First, we measured *discrimination accuracy*. We expected accuracy rates to be higher in the context of an upright face than in the context of an inverted face, as demonstrated by Vecera and Johnson (1995). Based on findings from Schwaninger, Lobmaier, and Fischer (2005), who reported comparable gaze location judgments when whole faces or the isolated eye regions were presented, one could probably expect accuracy rates to be similar for upright faces and eyes with the facial context removed. On the other hand, Langton et al. (2004) showed impaired gaze discrimination ability when the facial context information was removed as compared to eyes presented within the context of an upright face. In line with these latter results, one could thus also expect gaze discrimination ability to be reduced when eyes are presented without any facial context information.

Given that both looking at somebody and looking away from somebody are socially relevant signals that can indicate the intention of another person towards the perceiver, it is useful to be sensitive to both direct and averted gaze cues even in situations where the gaze stimulus is perceived only shortly. However, in the light of the great number of studies demonstrating enhanced representation and processing of direct gaze (Böckler et al., 2014; Boyer & Wang, 2018; Chen & Yeh, 2012; Farroni et al., 2002; Senju et al., 2005; Stein et al., 2011), we originally hypothesized that direct gaze processing is associated with higher accuracy rates compared to averted gaze in the present backward masking paradigm. This hypothesis is further affirmed by results suggesting a response bias toward direct gaze under conditions of uncertainty (Mareschal, Calder, Dadds, & Clifford, 2013; Mareschal, Otsuka, & Clifford, 2014)

Reviewing current findings on the processing of gaze did not allow us to come to a clear expectation about an interaction of context and gaze (especially with respect to the upright vs. inverted face context) under conditions of near-threshold stimulus presentation for two reasons. First, available evidence on the prioritized processing of direct (vs. averted) gaze in the context of upright (vs. inverted) face stimuli mostly relied on other experimental designs and dependent measures (e.g., response times rather than accuracy rates). Second, these response time (RT) studies reported the presence or absence of an interaction pattern depending on the paradigm used. For example, Senju et al. (2005) observed the absence of a direct-gaze advantage for inverted faces under conditions of conscious gaze processing. Similarly, Böckler, van der Wel, and Welsh (2015) reported the absence of attention capture by direct gaze when faces were presented upside-down. Others, however, showed that the preferential processing of direct gaze was independent of face inversion for unconscious processing of gaze (Chen & Yeh, 2012; Stein et al., 2011).

As a second behavioral measure, we analyzed RTs. Since participants were instructed to respond as quickly and accurately as possible, potential systematic differences in RTs across conditions, viewed in conjunction with accuracy rates, might provide insight into cognitive processes underlying the observed result patterns (e.g., with respect to a speed-accuracy tradeoff).

Data were also analyzed by means of *signal detection theory* (SDT), focusing on the sensitivity parameter *d’* and the response criterion *c* (see *Method* section of Experiment 1 for a detailed explanation). Based on Vecera and Johnson (1995) we expected gaze discrimination to be more efficient in upright faces than in inverted faces or for eyes only stimuli. We had no prior hypothesis about differences in gaze discrimination between inverted face and eyes-only stimuli. Note that the SDT analysis only reflects a measure of participants’ ability to discriminate direct from averted gaze in a given facial context (upright vs. inverted vs. eyes only) without allowing for any direct conclusion about which gaze direction is associated with a processing advantage (see Langton et al., 2004, and Vecera & Johnson, 1995, for a similar procedure).

Finally, we included a confidence measure after each trial to explore whether participants’ confidence in their performance mirrors actual discrimination accuracy. Dissociations between the subjective confidence and the objective performance measure may be informative with respect to potential origins of observed effects.

To anticipate the most surprising results of Experiment 1, we observed a clear advantage of averted gaze (in terms of faster RTs and higher accuracy rates for averted vs. direct gaze direction) for upright-face and eyes-only stimuli. Based on this surprising finding, we decided to run follow-up experiments in order to substantiate the evidence for an averted-gaze advantage. Furthermore, these follow-up experiments were also designed to systematically investigate potential boundary conditions of the averted-gaze advantage as observed in Experiment 1. More specifically, we tested whether the results of Experiment 1 are reliable (Experiment 2) and whether low-level task-specific demands provide a potential explanation for the observed results (Experiment 3). To anticipate our results once more, the results of Experiment 1 were closely replicated across experiments independent of the parameters varied, suggesting that the averted-gaze advantage as well as the declining ability to discriminate direct from averted gaze in upright faces versus inverted faces and eyes-only stimuli as observed in Experiment 1 is robust.

## Experiment 1

Experiment 1 investigated the ability of humans to discriminate direct from averted gaze direction in varying face contexts. To do so, targets with direct or averted gaze were briefly presented in a gaze-discrimination task and masked afterwards. Participants were asked to indicate the perceived gaze direction as quickly and accurately as possible via keypress.

The present setup implemented a backward-masking paradigm, a well-established way to study recognition of briefly presented target stimuli (see Breitmeyer & Ogmen, 2000, for a review). In backward-masking paradigms, the short presentation of a target stimulus is immediately followed by the presentation of a mask. This technique allows for the control of visual afterimages by accurately controlling the time available for sensory processing of the target stimulus. As one of the first, the study of Esteves and Öhmann (1993) used the backward masking procedure to investigate the recognition of emotional face expression as a function of different parameters. Their main result was that the stimulus-onset asynchrony (SOA) was the parameter that influenced recognition most. Further, backward masking can be applied to examine stimulus processing along different levels of visual consciousness by varying the presentation duration of the target stimulus. EEG backward-masking studies, for instance, investigated the time course of emotional face processing at subliminal and supraliminal processing stages (Eimer, Kiss, & Holmes, 2008; Kiss & Eimer, 2008; Vukusic, Ciorciari, & Crewther, 2017). Interestingly, several findings have shown that the modulation of affective responses in response to emotional stimuli and affective faces was similar for both masked stimuli and stimuli at longer presentation duration (e.g., Del Zotto & Pegna, 2015; Ruiz-Padial, Mata, Rodríguez, Fernández, & Vila, 2005; Ruiz-Padial & Vila, 2007). Relatedly, it has been shown that masked gaze cues are also capable of producing a gaze-cueing effect given that participant’s task set is tuned to the eye gaze information of the masked stimulus (Al-Janabi & Finkbeiner, 2012; Sato, Okada, & Toichi, 2007; but see Al-Janabi & Finkbeiner, 2014). Together, the results of these studies suggest that masked stimuli can produce qualitatively similar effects as have been reported for unmasked stimuli.

### Method

#### Participants

Twenty-five participants (mean age: 20.3 years, *SD* = 2.4 years, age range: 18–30 years, one male) took part in the study in exchange for course credit or payment. All participants reported normal or corrected-to-normal vision and gave informed consent prior to the study. On a nine-point scale ranging from 1 (“not difficult at all”) to 9 (“extremely difficult”), participants rated the task as rather difficult with an average of 7.0 points (*SD* = 1.7). A power analysis with G*Power (Faul, Erdfelder, Lang, & Buchner, 2007) was conducted based on a pilot study which incorporated stimulus material that differed only slightly from the stimulus material used in the present study. The power analysis indicated a sample size of 13 participants as sufficient to observe effects of face inversion, and a sample size of eight participants as sufficient to observe effects of gaze direction on accuracy (power = .95, α = .05). However, to account for possible dropouts and to avoid an underpowered study, we decided to recruit a minimum of 24 participants per experiment.

#### Apparatus and stimuli

**Fig. 1:**
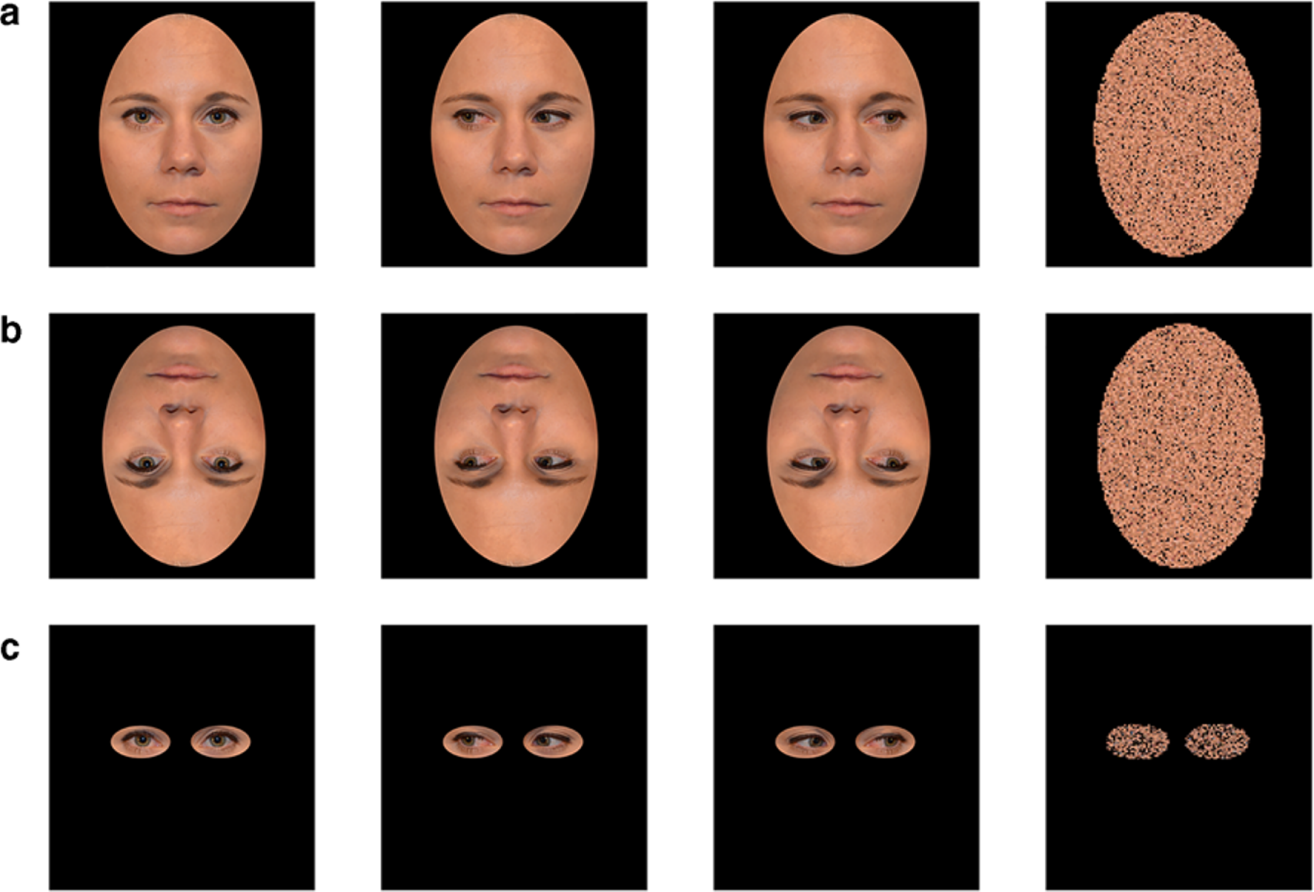
Stimulus material used throughout all experiments showing stimuli in (**A**) upright, (**B**) inverted, and (**C**) eyes-only condition with different gaze conditions (from left to right): direct gaze, left gaze, and right gaze, and the respective box-scrambled masks

In a dimly lit room, participants were seated in front of a 20-in. cathode ray monitor (temporal resolution, 100 Hz, spatial resolution 1,024 × 768 pixels) at a viewing distance of 65 cm. The experiment was programmed using Experiment Builder (SR Research, Ontario, Canada). Color face photographs of two female and two male Caucasian models that were taken by our lab served as stimulus material. All faces were of neutral facial expression and available with direct and averted gaze (to left and right). Gaze directions were inserted into the same face picture using photo editing software to maximize control. The faces were cropped with an oval shape, removing all external features (5.7° × 8.4° of visual angle, width × height). Stimuli were available in three versions, that is, as upright stimuli, inverted stimuli (by turning them upside down), and eyes in isolation (by cutting the eye regions of the upright-face stimuli into two separate ellipses with a size of 2.2° × 1.3° each, see Fig. 1).

Masks were created with MATLAB (Version R2017b) by dividing the direct gaze face of each of the four models into boxes of 20 × 20 pixels and randomly rearranging each box within the outline of the face (box scrambling). Using box-scrambled images as masks provided the advantage of creating unrecognizable stimuli without altering any low-level stimulus features (e.g., power spectrum, contrast, and luminance). One mask was created for each model and context condition, resulting in twelve different masks. Finally, we ensured that the relevant eye region always appeared at the same screen location for all stimulus conditions. Since eyes are not exactly centered in the head this resulted in slightly different stimulus positions for upright and eye stimuli as compared to inverted stimuli. The same location adjustment was done for the respective masks.

#### Procedure

**Fig. 2:**
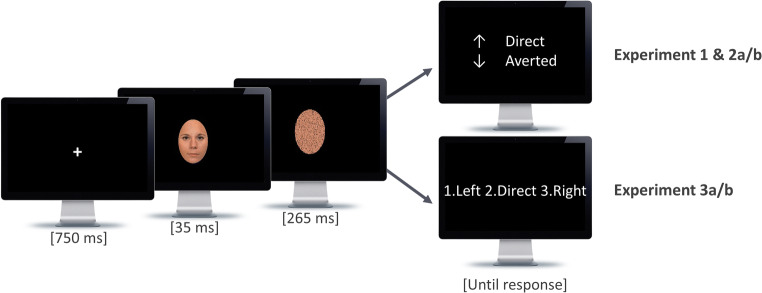
Schematic illustration of a trial. After the presentation of a fixation screen for 750 ms, the fixation cross was replaced by the target gaze stimulus (presentation duration: 35 ms) that was masked afterwards by a scrambled version of the respective stimulus (presentation duration: 265 ms). After that, a response screen was displayed which required pressing the key which corresponded to the gaze direction of the previously presented target stimulus as quickly and accurately as possible. Note that the response screen was different with respect to the response options displayed, depending on the design of the respective experiment. Following the response, participants were required to indicate the level of confidence associated with their response (not displayed in Fig. 2, see section *Procedure* for further details)

Before the experiment started, participants were instructed and familiarized with the different stimulus conditions. Each trial started with the presentation of a white central fixation cross (0.4° of visual angle, 700-ms presentation duration) on a black background, followed by a target stimulus with either direct or averted gaze direction for 35 ms. Shortly thereafter, the target was replaced by a mask, which was created based on the respective target stimulus, for 265 ms, resulting in a total presentation time of 300 ms (see Fig. 2). After that, the two response alternatives (arrow up vs. down) appeared on the screen together with the associated gaze direction (direct vs. averted). Participants indicated their response via keypress (up or down arrow key). For half of the participants, a direct gaze response was assigned to the “arrow-up” key, and an averted gaze response to the “arrow-down” key. For the other half, the response-key mapping was reversed. There was no time constraint with respect to the keypress, and the next display appeared only after the participant’s response. Participants received the instruction to respond as accurately and fast as possible, and to guess in case they were not able to detect any direction. Participants provided a confidence rating after each trial. To do so, they entered a number on the computer keyboard using a rating scale which ranged from one to five (with one meaning “definitely not detected” and five meaning “definitely detected”). The next trial started 500 ms after participants entered their confidence rating. Stimuli were presented in a fully randomized order in ten blocks of 48 trials each (resulting in 480 trials in total) with breaks in between the blocks. Each stimulus condition and gaze condition appeared equally often. The averted gaze direction (left vs. right) was counterbalanced across participants such that each observer only saw one averted gaze direction across all trials. This design decision was made as part of a strategy that applies to the entire series of presented experiments (for a strategy overview, see Table 1). Among other things, we varied between experiments whether averted gaze direction included only one averted gaze direction per participant, or both (i.e., averted gaze targets to the left and right direction). At the end of the experiment, participants judged the overall difficulty of the task on a scale ranging from one (“not difficult at all”) to nine (“extremely difficult”). This difficulty measure was included in order to obtain an initial assessment of the subjectively experienced difficulty of a newly designed task but served no further diagnostic function within the experimental process.

**Table 1:** Overview of the experimental manipulations across experiments

		Two response options		Three response options	
		Stimulus ratio	Stimulus ratio	Stimulus ratio	Stimulus ratio
		1 : 1 : 1	1 : 0.5 : 0.5	1 : 1 : 1	1 : 0.5 : 0.5
		(direct/left/right)	(direct/left/right)	(direct/left/right)	(direct/left/right)
Target-key mapping for averted gaze	1 : 1	Experiment 1^a^		Experiment 3a	Experiment 3b
	2 : 1	Experiment 2a	Experiment 2b		

*Note.* Stimulus ratio refers to the proportions at which direct versus left versus right targets have been displayed in each experiment indicating that *each target* was presented at equal proportion (1:1:1) or that *direct* and *averted* gaze targets (summed up for left and right gaze) were displayed at equal proportion

^a^In Experiment 1, there were only *two* different targets (direct/averted gaze), while the averted gaze targets were of the same direction within each participant. Consequently, Experiment 1 is an exception to the stimulus ratio classification as listed in the table above: Presenting an equal proportion of each target automatically comes along with an equal proportion of direct and averted gaze targets

#### Design and analyses

We measured accuracy rates (percentage of correct responses) and confidence ratings. Response time was defined as the interval from the onset of the response screen until the first key press. For the analysis of RTs, we eliminated all error trials (19.87% of the data). After removing error trials, we additionally excluded trials with anticipatory responses or extremely high RTs (RTs < 100 ms or RTs > 5,000 ms; corresponding to 0.28% of the remaining data). After that, an outlier correction was applied by removing trials with RTs that deviated more than 2.5 SDs from the corresponding cell mean (calculated separately per participant and condition), corresponding to 3.25% of the remaining data. Separate repeated-measures analyses of variance (ANOVAs, α = .05, throughout) with context (upright vs. inverted vs. eyes only) and gaze (direct vs. averted) as within-subjects factors were conducted to analyze accuracy rates, confidence ratings, and RTs. Significant interactions were further analyzed by using additional one-way repeated-measures ANOVAs. Two-tailed paired-samples *t*-tests were conducted for follow-up comparisons between conditions. For ANOVAs and paired-samples *t*-tests we report *ƞ*^2^_p_ and Cohen’s *d*_*z*_ (calculated as ${d}_z=\frac{t}{\sqrt{n}}$) as effect size estimates, respectively. Sphericity violations were assessed using the Mauchly’s sphericity test. In case of sphericity violations, we report Greenhouse-Geisser corrected *p* values along with original degrees of freedoms as well as corresponding *ε* values. To explore whether the averted-gaze advantage was merely driven by a response bias under high uncertainty, we additionally analyzed accuracy rates including high confidence trials only (i.e., the upper 50% of confidence ratings). Note that while we report the analysis of confidence ratings in the results section of each experiment, we will restrict the combined interpretation of these analyses to the General Discussion.

As an additional estimate of gaze discrimination performance, we computed the sensitivity parameter *d’* (*d’*= *z*(hit rate) – *z*(false alarm rate)) and the response criterion *c* (*c* = -0.5 [*z*(hit rate) + *z*(false alarm rate)]) separately for each participant for each stimulus condition as specified by signal detection theory (SDT) and implemented by Macmillan and Creelman (2010). That is, the *d’* score measured the sensitivity of subjects to distinguish between direct and averted gaze in the context of an upright face, an inverted face, and for the eyes-only condition. Direct gaze trials were assigned to represent signal trials, while averted gaze trials served as noise trials. To avoid infinite *d’* values in case of perfect accuracy we adjusted proportions of 0 and 1 to 1/(2N) and 1-1/(2N), respectively, where N reflects the number of trials on which the proportion is based (Macmillan & Creelman, 2010). The response criterion *c* reflects the participants’ tendency toward one or the other response (Macmillan & Creelman, 2010) with more positive values indicating a higher tendency toward “averted” answers. We conducted repeated-measures one-way ANOVAs for *d’* and *c* with context (upright vs. inverted vs. eyes) as within-subjects factor.^1^ Two-tailed pairwise t-tests were conducted for follow-up comparisons between conditions. Note that none of the participants had to be excluded due to any *d’* lower than zero (indicating that the false alarm rate was higher than the hit rate, see Macmillan and Creelman, 2010).

### Results

#### Accuracy

**Fig. 3:**
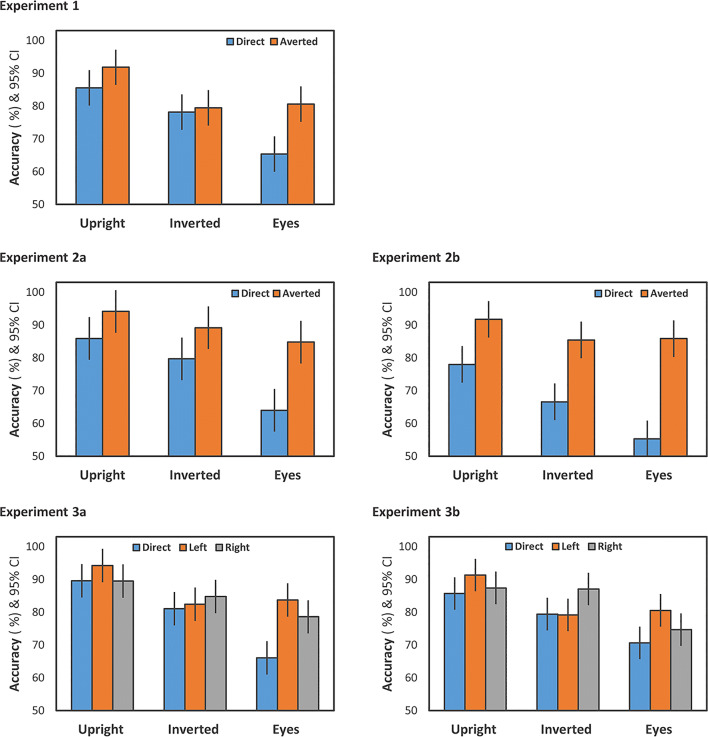
Accuracy as a function of context (upright vs. inverted vs. eyes only) and gaze (direct vs. averted (Experiment 1, 1a, and 2b); direct vs. left vs. right (Experiment 3a and 3b)). Error bars represent 95% confidence intervals (Loftus & Masson, 1994)

The effect of context, *F*(2,48) = 41.17, *p* < .001, *ƞ*^2^_p_ = .63, indicated differences in accuracy rate across stimuli (see Fig. 3). Accuracy rate was highest for upright faces, lower for inverted faces, and lowest for the eyes-only condition (see Table 2). Paired *t*-tests revealed significant pairwise differences between all context conditions (*p*s < .05). Also, there was an effect of gaze, *F*(1,24) = 6.74, *p* = .016, *ƞ*^2^_p_ = .22. Interestingly, and contrary to our initial assumption, accuracy was higher for averted gaze (*M* = 83.93%, *SE* = 2.29%) compared to direct gaze (*M* = 76.33%, *SE* = 2.99%). The interaction of context and gaze was not significant, *F*(2,48) = 3.44, *ε* = .70, *p* = .059, *ƞ*^2^_p_ = .13. Given the medium effect size, we further analyzed this interaction revealing a nominal tendency towards higher accuracy rates for averted gaze (vs. direct gaze) for both upright and eyes only stimuli, *t*(24) = 2.68*, p* = .013, *d* = 0.54, and *t*(24) = 2.50*, p* = .020, *d* = 0.50, respectively, while there was no such difference for inverted stimuli, *t*(24) = 0.37*, p* = .716, *d* = 0.07. Analyzing only high-confidence trials showed a very similar pattern of results, as revealed by the significant effect of context, *F*(2,48) = 18.52, *p* < .001, *ƞ*^2^_p_ = .44, the significant effect of gaze, *F*(1,24) = 5.13, *p* = .033, *ƞ*^2^_p_ = .18, and the significant interaction, *F*(2,48) = 5.35, *ε* = .80, *p* = .013, *ƞ*^2^_p_ = .18.

**Table 2:** Mean accuracy rates and response times (RTs) across experiments

	Upright		Inverted		Eyes only	
	*M*	*SE*	*M*	*SE*	*M*	*SE*
Experiment 1						
Accuracy rate (%)	88.65	1.84	78.78	2.71	72.96	2.68
Accuracy rate (direct) (%)	85.50	2.43	78.14	3.21	65.35	4.58
Accuracy rate (averted) (%)	91.80	1.90	79.42	3.22	80.58	3.46
RTs (ms)	733	41.0	842	49.6	863	52.4
RTs (direct) (ms)	750	47.0	850	51.7	908	57.8
RTs (averted) (ms)	716	40.8	834	52.4	819	55.5
Experiment 2a						
Accuracy rate (%)	89.99	1.86	84.41	2.44	74.39	2.49
Accuracy rate (direct) (%)	85.86	3.38	79.69	4.09	63.99	5.44
Accuracy rate (averted) (%)	94.12	1.16	89.14	1.76	84.78	3.14
RTs (ms)	705	37.1	783	36.9	882	51.1
RTs (direct) (ms)	728	45.0	803	40.5	978	72.4
RTs (averted) (ms)	682	35.6	762	42.0	787	45.6
Experiment 2b						
Accuracy rate (%)	84.84	1.90	75.99	2.17	70.55	1.83
Accuracy rate (direct) (%)	77.97	3.46	66.56	3.42	55.26	3.53
Accuracy rate (averted) (%)	91.72	1.70	85.42	3.08	85.83	1.89
RTs (ms)	771	41.3	871	50.5	895	48.7
RTs (direct) (ms)	833	49.1	913	47.8	948	57.6
RTs (averted) (ms)	708	38.5	828	62.4	843	47.0
Experiment 3a						
Accuracy rate (%)	91.07	2.41	82.74	3.02	76.11	2.78
Accuracy rate (direct) (%)	89.54	2.72	81.04	3.36	66.07	5.05
Accuracy rate (left) (%)	94.22	2.19	82.40	4.00	83.67	2.94
Accuracy rate (right) (%)	89.46	2.79	84.78	4.12	78.57	3.58
RTs (ms)	512	39.6	591	47.7	630	54.5
RTs (direct) (ms)	584	41.2	648	48.2	744	69.5
RTs (left) (ms)	504	43.6	585	51.9	585	48.2
RTs (right) (ms)	449	38.3	539	53.0	560	54.2
Experiment 3b						
Accuracy rate (%)	88.12	1.90	81.87	2.60	75.27	2.74
Accuracy rate (direct) (%)	85.65	2.36	79.40	2.87	70.60	4.40
Accuracy rate (left) (%)	91.30	2.02	79.13	4.06	80.54	3.84
Accuracy rate (right) (%)	87.39	2.11	87.07	3.50	74.67	4.05
RTs (ms)	538	26.4	618	30.3	635	33.8
RTs (direct) (ms)	586	30.0	658	35.8	725	52.4
RTs (left) (ms)	536	29.0	650	39.7	608	31.6
RTs (right) (ms)	493	26.3	546	31.9	572	37.1

*Note.* Mean (*M*) and standard error of the mean (*SE*) are depicted for accuracy rates and RTs separately for each context condition (upright vs. inverted vs. eyes only) and gaze condition (direct vs. averted (Experiments 1, 2a, and 2b); direct vs. left vs. right (Experiment 3a and 3b))

#### Response times

**Fig. 4:**
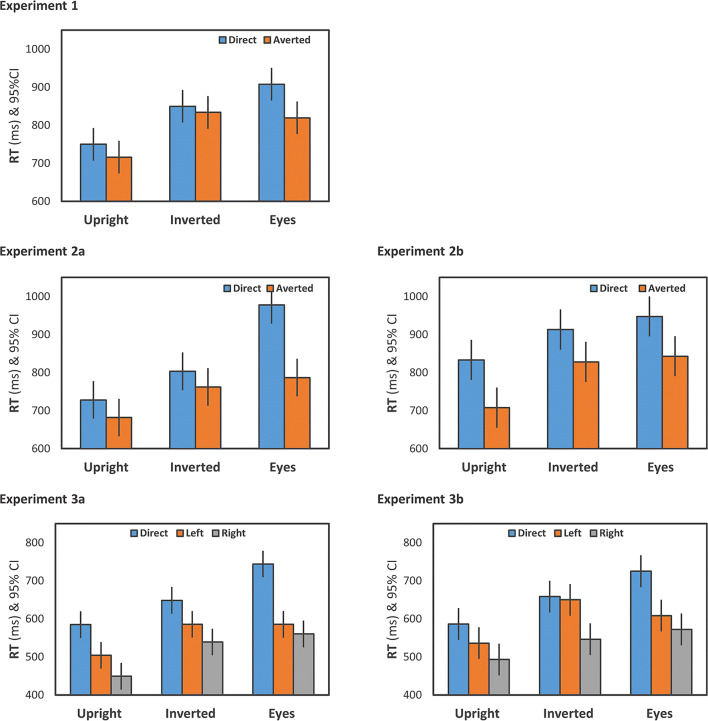
Response times (RTs) as a function of context (upright vs. inverted vs. eyes only) and gaze (direct vs. averted (Experiments 1, 1a, and 2b); direct vs. left vs. right (Experiment 3a and 3b)). Error bars represent 95% confidence intervals (Loftus & Masson, 1994)

Similar to the accuracy rate, we observed a significant main effect of context on RTs, *F*(2,48) = 14.78, *ε* = .76, *p* < .001, *ƞ*^2^_p_ = .38 (see Fig. 4). Participants responded faster in the upright condition compared to both the inverted condition and the eyes condition, both *p*s < .001. RTs did not differ between the inverted and the eyes-only condition, *t*(24) = 0.99*, p* = .332, *d* = 0.20 (see Table 2). The effect of gaze was not statistically significant, *F*(1,24) = 3.12, *p* = .090, *ƞ*^2^_p_ = .12, but indicated a nominal tendency towards faster RTs in the averted condition (*M* = 790 ms, *SE* = 46 ms) compared to the direct condition (*M* = 836 ms, *SE* = 49 ms). The interaction was not significant, *F*(2,48) = 1.57, *p* = .219, *ƞ*^2^_p_ = .06).

#### Signal-detection analyses

Participants differed in their discrimination sensitivity *d’* dependent on context, *F*(2,48) = 54.27, *p* < .001, *ƞ*^2^_p_ = .69. Specifically, they showed increased gaze discrimination sensitivity for upright stimuli (*M* = 2.88, *SE* = 0.22) compared to both inverted stimuli (*M* = 1.90, *SE* = 0.23) and the eyes-only condition (*M* = 1.58, *SE* = 0.22), *t*(24) = 7.92*, p* < .001, *d* = 1.58, and *t*(24) = 9.83*, p* < .001, *d* = 1.97, respectively. Importantly, gaze-discrimination sensitivity was also higher for inverted stimuli as compared to the eyes-only condition, *t*(24) = 2.37*, p* = .026, *d* = 0.47. Participants showed no difference in the response criterion *c* across context conditions, *F*(2,48) = 2.48, *ε* = .76, *p* = .110, *ƞ*^2^_p_ = .09. The mean response criterion *c* amounted to 0.19 (*SE* = 0.06) for the upright condition, to 0.05 (*SE* = 0.07) for the inverted condition, and to 0.26 (*SE* = 0.11) for the eyes-only condition, indicating a small tendency toward responding with “averted gaze”.

#### Response confidence

Analogous to accuracy rates, an effect of context emerged, *F*(2,48) = 45.43, *p* < .001, *ƞ*^2^_p_ = .65, with highest confidence ratings for upright faces (*M* = 3.74, *SE* = 0.17), followed by inverted faces (*M* = 3.22, *SE* = 0.17) and the eyes-only condition, (*M* = 2.85, *SE* = 0.20). Paired *t-*tests showed significant differences between all context conditions (*p*s < .001). Also in line with accuracy findings, the significant effect of gaze, *F*(1,24) = 9.79, *p* = .005, *ƞ*^2^_p_ = .29, was driven by higher confidence ratings for averted gaze (*M* = 3.38, *SE* = 0.18) compared to direct gaze (*M* = 3.16, *SE* = 0.17). The interaction was not statistically significant, *F*(2,48) = 0.54, *p* = .584, *ƞ*^2^_p_ = .02.

### Experiment 1: Discussion

Experiment 1 was designed to examine performance in a gaze-discrimination task for varying face contexts under near-threshold presentation durations using a backward-masking paradigm. We observed straightforward effects of context and gaze across measures. The effect of context revealed gradually decreasing accuracy rates for upright faces versus inverted faces versus eyes-only stimuli, in line with our predictions. Decreasing accuracy rates came along with gradually increasing RTs for upright face versus inverted face versus eyes-only stimuli (note that while the absolute values for RTs reflected this ordinal pattern, the difference in RTs between the inverted and eyes condition was actually not statistically significant). This ordinal pattern was also reflected in both the confidence rating and the sensitivity parameter *d*’ with decreasing sensitivity for upright face versus inverted face versus eyes-only stimuli (cf. Vecera & Johnson, 1995). Presumably, facial context information facilitates gaze discrimination, especially if humans are very familiar with the context provided, as in case of an upright presentation of faces. These effects of context cannot be explained by differences in response bias, as we did not find significant differences between response biases across conditions.

We observed an effect of gaze, but in the opposite direction as expected based on the abundance of evidence for a direct-gaze advantage in a variety of experimental research paradigms. Interestingly, we observed higher accuracy rates for averted versus direct gaze (for upright-face and eyes-only stimuli), and higher confidence as well as a tendency toward faster responses for averted versus direct gaze (across all context conditions). Note that accuracy rates were highest in conditions where participants responded fastest, thereby ruling out the presence of a speed-accuracy tradeoff.

Consequently, we asked ourselves about the reasons for the currently observed surprising averted-gaze advantage. Does it truly represent a processing advantage for averted (vs. direct) gaze when targets are presented near-threshold, that is, with a certain degree of uncertainty about the sensory input, or are there any confounds inherent to the task demands that modulate the gaze effect (see e.g., Burra, Mares, & Senju, 2019, for a review on the influence of task demands on gaze processing)? The former account contrasts with results of several previous studies demonstrating that the perceived gaze direction was biased toward *direct* as uncertainty about the visual input increased (e.g., by adding noise or removing facial context information; see Mareschal et al., 2013, 2014). The latter account could be motivated by the following facts: First, in Experiment 1 we opted for a two-alternative forced-choice task in order to be able to distinguish between perceptual sensitivity and response bias according to signal detection theory. Consequently, each participant had to decide whether targets displayed direct or averted gaze, but the direction of the averted gaze (to the left or right) was held constant within participants, while the specific pairing (direct with left gaze or direct with right gaze) was counterbalanced across participants. Second, direct gaze, which has been implemented in terms of straight gaze in the present study, is characterized by symmetry on low-level perception: Pupil and iris are centrally arranged within the eye region and surrounded by the white sclera. In contrast, pupil and iris shift to the left or right edge of the eye region for averted gaze, yielding a larger coherent area of eye white. We consider it plausible that the observed effects of gaze are due to the increased saliency of the eye white for averted (vs. direct) gaze targets. Under conditions of near-threshold presentation duration, the salient eye white of the averted gaze might pop out, while the eye white and the pupil become somewhat “blurred” for direct gaze, potentially making it harder to indicate gaze direction. We conducted the following series of experiments in order to test whether the results of Experiment 1 are reliable (Experiment 2) and to rule out low-level stimulus-specific demands as described above as a potential explanation of the present results (Experiment 3).

In these follow-up experiments, each participant was confronted with gaze stimuli of both left *and* right averted gaze direction. However, participants still had to classify gaze stimuli as direct versus averted gaze (two response options) in Experiment 2. Consequently, the target-key mapping for averted gaze targets was at a ratio of 2:1 (i.e., direct gaze was mapped onto one key and both averted-gaze conditions were mapped onto the other key) in Experiment 2. In Experiment 3, participants were required to distinguish between direct, left, and right gaze (three response options); therefore, the target-key mapping was again at equal ratio (i.e., direct gaze was mapped onto one key, left gaze onto another key, and right gaze onto a third key) in Experiment 3. Another experimental manipulation was motivated by the fact that each target (direct vs. averted) was presented at the same ratio, and therefore, both direct and averted gaze direction were presented in 50% of the trials in Experiment 1. With three gaze directions as stimuli (as in Experiments 2 and 3) it was not possible to maintain both an equal ratio of target presentation and an equal ratio of direct versus averted gaze targets (summed up for left and right gaze) at the same time. More precisely, as soon as each gaze direction (direct vs. left vs. right) will be presented in one third of the trials, the proportion of direct versus averted gaze targets is not equal anymore but will shift to 1:2 for direct versus averted gaze direction. To address this issue, we manipulated the relative target presentation frequency, that is, whether targets of each gaze condition (direct vs. left vs. right) were presented at equal proportion, or whether direct and averted gaze targets were presented in half of the trials with the averted-gaze condition (with an equal number of left and right gaze stimuli). Table 1 provides an overview of these experimental manipulations across experiments.

## Experiment 2

Experiment 2 was a conceptual replication of Experiment 1 and was conducted to test whether the averted-gaze advantage as observed in Experiment 1 is reliable. We adapted our task in a way that each participant was not only presented with averted gaze targets of the same direction (left or right) but with averted gaze targets signaling both gaze directions (left and right). As in Experiment 1, there were only two response options indicating direct vs. averted gaze. Consequently, the target-key mapping for averted gaze changed from 1:1 in Experiment 1 to 2:1 in Experiment 2 (left and right averted gaze targets were mapped onto the same key). If, for example, the averted-gaze advantage was mainly due to the salient sclera in the averted gaze targets, as hypothesized above, we would expect to observe the same result pattern as in Experiment 1, since the task of Experiment 2 required the same classification of direct and averted gaze as in Experiment 1, but no differentiation between left and right gaze.

In Experiment 1, presenting both targets at equal ratio can be equated with presenting an equal proportion of direct and averted gaze targets. However, the design of Experiment 2 entailed that both types of equalities cannot be maintained at the same time: If all three targets (direct/left/right) were presented at equal ratio, the ratio of presenting direct and averted gaze targets would inevitably change from equal ratio to 1:2 (for direct/averted gaze targets), and vice versa. Therefore, comparability between Experiments 1 and 2 would be reduced. In order to rule out that the ratio of stimulus presentation modulates the effect, we therefore conducted two versions of this experiment (Experiment 2a and 2b). While each target (direct vs. left vs. right) was presented at equal ratio in Experiment 2a, the ratio of target presentation was altered in Experiment 2b in order to present direct versus averted gaze targets in half of the trials each (see Method section for details). Again, if the averted-gaze advantage was due to the salient sclera in the averted gaze targets, we would expect to observe the same result pattern as in Experiment 1 across both Experiment 2a and 2b, independent of the ratio of target presentation.

### Experiment 2A

#### Method

##### Participants

We tested 24 new participants (mean age: 24.2 years, *SD* = 4.1 years, age range: 19–39 years, two male). No participant had to be excluded (all individual *d*s > 0). Participants reported normal or corrected-to-normal vision and received monetary compensation for participation. They provided written informed consent before the experiment started. On average, the perceived task difficulty amounted to 4.6 points (*SD* = 2.1) on the same 9-point scale as used in the previous experiment.

##### Apparatus, stimuli, and procedure

Technical equipment, stimulus material, and procedure were identical to Experiment 1. The only difference was that instead of counterbalancing the averted gaze direction (left vs. right) across participants, we presented both averted gaze directions within each participant. That is, participants were not required to distinguish between left- and rightward-averted gaze via response key (since these two response options were mapped onto a single key for averted gaze), but were still presented with all three gaze directions (direct vs. left vs. right). Stimuli of each gaze direction (direct vs. left vs. right) were displayed at equal ratio in Experiment 2a. Stimuli were presented in a fully randomized order in nine blocks of 56 trials each (resulting in 504 trials in total) with breaks in between blocks.

##### Design and analyses

We conducted the same analyses as in Experiment 1. In Experiment 2a, for the analysis of RTs, we eliminated 14.93% of the data due to incorrect responses, then another 0.46% of the data due to anticipatory responses (RTs < 100 ms) or extremely long RTs (RTs > 5,000 ms), and, finally, 3.24% of the data due to outlier correction (RTs > 2.5 SDs from the corresponding cell mean; calculated separately per participant and condition).

#### Results

##### Accuracy

As in Experiment 1, accuracy significantly differed between context conditions, *F*(2,46) = 51.47, *p* < .001, *ƞ*^2^_p_ = .69 (see Fig. 3), with highest accuracy rates for upright faces, lower accuracy rates for inverted faces, and lowest accuracy rates in the eyes-only condition (*p*s < .001 for paired *t*-tests) (see Table 2). Again, the effect of gaze revealed higher accuracy rates for averted (89.35%, *SE* = 1.53%) compared to direct gaze (76.51%, *SE* = 3.62%), *F*(1,23) = 12.40, *p* = .002, *ƞ*^2^_p_ = .35. The interaction was not significant, *F*(2,46) = 2.30, *ε* = .62, *p* = .136, *ƞ*^2^_p_ = .09. Analyzing high-confidence trials only revealed a similar pattern of results, as demonstrated by the significant effect of context, *F*(2,46) = 17.58, *ε* = .81, *p* < .001, *ƞ*^2^_p_ = .43, and the significant effect of gaze, *F*(1,23) = 10.04, *p* = .004, *ƞ*^2^_p_ = .30. The interaction was significant, *F*(2,46) = 5.44, *ε* = .68, *p* = .018, *ƞ*^2^_p_ = .19, revealing gaze effects in both the inverted and eyes only context condition, while there was no such effect in the upright context condition.

##### Response times

Participants responded fastest in the upright condition, slower in the inverted condition, and slowest in the eyes-only condition, *F*(2,46) = 25.09, *p* < .001, *ƞ*^2^_p_ = .52 (see Fig. 4). Pairwise *t*-tests indicated significant differences across all three conditions (all *p*s < .05) (see Table 2). In line with accuracy data, we observed faster RTs for averted gaze (743 ms, *SE* = 39 ms) compared to direct gaze (836 ms, *SE* = 48 ms), *F*(1, 23) = 6.13, *p* = .021, *ƞ*^2^_p_ = .21. The significant interaction, *F*(2,46) = 6.01, *ε* = .61, *p* = .016, *ƞ*^2^_p_ = .21, indicated that the difference between direct and averted gaze was significant for the eyes-only condition, *t*(23) = 2.94, *p* = .007, *d* = 0.60, while there was no such significant difference in the upright, *t*(23) = 1.41, *p* = .171, *d* = 0.29, or the inverted condition, *t*(23) = 1.12, *p* = .274, *d* = 0.23.

##### Signal-detection analyses

Participants differed in their sensitivity *d’* dependent on context, *F*(2, 46) = 39.51, *p* < .001, *ƞ*^2^_p_ = .63, with highest sensitivity toward upright faces (*M* = 2.95, *SE* = 0.17), lower sensitivity toward inverted faces (*M* = 2.40, *SE* = 0.21), and lowest sensitivity toward stimuli of the eyes-only condition (*M* = 1.78, *SE* = 0.20). Paired *t*-tests indicated significant pairwise differences between all context conditions (*p*s < .001). There was no difference in response criteria depending on context condition, *F*(2, 46) = 2.02, *ε* = .66, *p* = .162, *ƞ*^2^_p_ = .08, with a *c* of 0.22 (*SE* = 0.06) for the upright condition, of 0.17 (*SE* = 0.07) for the inverted condition, and of 0.41 (*SE* = 0.14) for the eyes-only condition. These positive values of *c* indicate a small response bias towards responding with “averted gaze”.

##### Response confidence

Confidence ratings reflected the same result pattern as reported for the previous experiment: We observed a main effect of context, *F*(2,46) = 53.37, *p* < .001, *ƞ*^2^_p_ = .70, but no effect of gaze, *F*(1,23) = 3.60, *p* = .071, *ƞ*^2^_p_ = .14. The interaction was not significant, *F*(2,46) = 2.14, *p* = .130, *ƞ*^2^_p_ = .09.

### Experiment 2B

#### Method

##### Participants

We recruited 25 new participants. Data of one participant had to be excluded due to technical failure when saving the data file; no other participant had to be excluded (all individual *d*s > 0). The data of the remaining 24 participants were analyzed (mean age: 24.9 years, *SD* = 3.6 years, age range: 20–36 years, 7 male). All participants received monetary compensation for participation, reported normal or corrected-to-normal vision and gave written informed consent before participation. On average, the perceived task difficulty amounted to 5.7 points (*SD* = 1.8) on the same 9-point scale as used in the previous experiments.

##### Apparatus, stimuli, and procedure

In Experiment 2b, we used the identical technical equipment and stimulus material as in the previous experiments. The study design was mostly identical to Experiment 2a. We only changed the relative frequency at which stimuli of each gaze direction were displayed. Instead of displaying each gaze direction (direct vs. left vs. right) at equal ratio (like in Experiment 2a), direct gaze stimuli were presented in 50% of the total number of trials, while the averted gaze stimuli were either looking to the left or right at the same ratio for the other 50% of trials. Still, participants were not required to distinguish between left- and rightward-averted gaze via response keys, since these two response options were mapped onto a single key for averted gaze. Stimuli were presented in a fully randomized order in ten blocks of 48 trials each (resulting in a total of 480 trials) with breaks in between blocks.

##### Design and analyses

We conducted the same analyses as in the previous Experiment. For the analysis of RTs, we removed 22.87% of the data set due to incorrect responses. After that, 0.45% of the data were eliminated due to anticipatory responses (RTs < 100 ms) or extremely high RTs (RTs > 5,000 ms), and, finally, 3.49% of the data due to outlier correction (RTs > 2.5 SDs from the corresponding cell mean; calculated separately per participant and condition).

#### Results

##### Accuracy

The repeated-measures ANOVA revealed an effect of context, *F*(2,46) = 44.38, *p* < .001, *ƞ*^2^_p_ = .66 (see Fig. 3). Accuracy rates were highest for upright faces, lower for inverted faces, and lowest for the eyes-only condition (see Table 2). Paired *t*-tests showed significant differences between all stimulus conditions (*p*s < .05). Accuracy rates were higher for the averted-gaze condition (*M* = 87.66%, *SE* = 1.74%) compared to the direct gaze condition (*M* = 66.60%, *SE* = 2.76%), *F*(1,23) = 49.50, *p* < .001, *ƞ*^2^_p_ = .68. The significant interaction of context and gaze, *F*(2,46) = 4.84, *ε* = .69, *p* = .025, *ƞ*^2^_p_ = .17, indicated that the difference in accuracy rates between direct and averted gaze was greatest for the eyes-only condition, *t*(23) = 7.06, *p* < .001, *d* = 1.44, and smaller for both the upright, *t*(23) = 3.52, *p* < .002, *d* = 0.72, and the inverted context condition, *t*(23) = 3.88, *p* = .001, *d* = 0.79. We further broke down this interaction by conducting separate repeated-measures ANOVAs for each gaze direction condition. For direct gaze, the effect of context, *F*(2,46) = 19.50, *p* < .001, *ƞ*^2^_p_ = .46, revealed significant differences across all three context conditions (*p*s < .05). For averted gaze, context had no significant influence on accuracy rate, *F*(2,46) = 3.63, *ε* = .63, *p* = .058, *ƞ*^2^_p_ = .14. The analysis of high-confidence trials only left the overall result patterns unchanged, as demonstrated by the significant effect of context, *F*(2,46) = 19.22, *p* < .001, *ƞ*^2^_p_ = .43, the significant effect of gaze, *F*(1,23) = 35.76, *p* < .001, *ƞ*^2^_p_ = .61, and the significant interaction, *F*(2,46) = 6.11, *ε* = .68, *p* = .012, *ƞ*^2^_p_ = .21.

##### Response times

Analyzing RTs revealed a significant effect of context, *F*(2,46) = 14.09, *p* < .001, *ƞ*^2^_p_ = .38 (see Fig. 4). Paired *t*-tests revealed that participants initiated the response key press faster in the upright condition compared to both the inverted condition and the eyes-only condition (both *p*s < .001). RTs did not differ between the inverted condition and the eyes-only condition, *t*(23) = 0.87, *p* = .391, *d* = 0.18 (see Table 2). The effect of gaze was significant, *F*(1,23) = 17.54, *p* < .001, *ƞ*^2^_p_ = .43, with faster RTs in the averted condition (*M* = 793 ms, *SE* = 45 ms) compared to the direct condition (*M* = 898 ms, *SE* = 48 ms). The interaction was not significant, *F*(2,46) = 0.29, *ε* = .74, *p* = .683, *ƞ*^2^_p_ = .01.

##### Signal-detection analyses

The discriminatory sensitivity index *d*’ significantly differed across context, *F*(2, 46) = 50.80, *p* < .001, *ƞ*^2^_p_ = .69. Participants showed highest sensitivity toward upright faces (*M* = 2.50, *SE* = 0.19) compared to both inverted faces (*M* = 1.72, *SE* = 0.16) and the eyes-only condition (*M* = 1.32, *SE* = 0.13). Paired *t*-tests indicated significant differences in sensitivity between all context conditions (*p*s < .05). Participants did not differ in their response criteria across context conditions, *F*(2, 46) = 2.01, *ε* = .76, *p* = .158, *ƞ*^2^_p_ = .08. The mean response criterion *c* amounted to 0.33 (*SE* = 0.08) for the upright condition, to 0.38 (*SE* = 0.09) for the inverted condition, and to 0.52 (*SE* = 0.07) for the eyes-only condition, indicating a small response bias towards responding with “averted gaze”.

##### Response confidence

The analysis of confidence ratings showed highest confidence ratings for upright faces (*M* = 3.74, *SE* = 0.15), followed by inverted faces, (*M* = 3.34, *SE* = 0.16), and lowest confidence ratings for the eyes-only condition (*M* = 2.94, *SE* = 0.17), *F*(2,46) = 24.06, *ε* = .66, *p* < .001, *ƞ*^2^_p_ = .51. Paired *t*-tests indicated significant differences across all context conditions (*p*s < .05). Participants reported higher confidence in their ratings in the averted-gaze condition (*M* = 3.49, *SE* = 0.14) compared to the direct gaze condition (*M* = 3.19, *SE* = 0.16), *F*(1, 23) = 30.26, *p* < .001, *ƞ*^2^_p_ = .57. The interaction was not significant, *F*(2,46) = 0.10, *p* = .908, *ƞ*^2^_p_ = .51.

### Experiment 2: Discussion

The results of Experiment 2a and 2b closely mirrored the findings of Experiment 1: We observed that accuracy rates and sensitivity gradually decreased from the upright-face condition, via the inverted-face condition, to the eyes-only condition, while RTs followed the reversed pattern. These results confirmed the ordinal result pattern as observed in Experiment 1. Regarding the effect of gaze, both experiments (2a and 2b) demonstrated that participants’ performance (in terms of accuracy and RTs) was better for targets displaying averted gaze compared to targets displaying direct gaze. Again, this averted-gaze advantage is consistent with the observations in Experiment 1. The averted-gaze advantage in RTs was predominantly present in the eyes-only condition (as indicated by the significant interaction of context and gaze in Experiment 2a), and the context effect in accuracy rates was predominantly pronounced for direct gaze (as indicated by the significant interaction of context and gaze in Experiment 2b). Interestingly, these observations demonstrate that both the averted-gaze advantage (as reflected in RTs) and the context effect (as reflected in accuracy rates and RTs) are rather robust for those conditions where our data indicate impaired gaze discrimination performance, namely when targets show direct gaze or when the isolated eye region is presented. This result differs from the corresponding pattern in Experiment 1, where the interaction of gaze direction and context was neither significant for accuracy rates nor for RTs (see *General discussion*). Taken together, the present results suggest that both the context effect and the averted-gaze advantage are reliable phenomena. We further conclude that changes in the relative frequency of target distribution left the overall pattern of results intact, given that the task always required discriminating between direct and averted gaze targets, irrespective of the direction of the averted gaze.

While Experiment 2 included stimuli looking into both left and right directions, participants were still not required to distinguish between left and right gaze direction by pressing dedicated keys in this setting. Thus, we considered it possible that the effects observed in Experiments 1 and 2 might vanish if gaze discrimination between left and right gaze direction is required. Experiment 3 was conducted to explicitly test this possibility.

## Experiment 3

Experiment 3 was conducted to test whether the response format has driven the averted-gaze advantage observed in Experiments 1 and 2. In order to do so, we slightly adapted our design. Experiment 1 required participants to distinguish between direct and averted gaze, while each participant was only presented with one of the two possible averted gaze directions. In Experiment 2, each participant was presented with both types of averted gaze (left *and* right direction). However, still no judgment about the direction of the averted gaze was required. In Experiment 3, we now introduced a third response key, so that each target (direct vs. left vs. right) was allocated to one response key. Consequently, the task was expanded in a way that participants also had to discriminate between left and right gaze direction. We argued that if participants discriminated between direct and averted gaze based on the brightness of the eye region, which is brighter in the averted-gaze condition due to the larger visible area of eye white, the averted-gaze advantage as observed in Experiments 1 and 2 should diminish or even reverse when participants are required to additionally differentiate between left and right gaze direction. More specifically, participants should still able to detect the salient eye white of the averted gaze targets with high certainty but should experience great difficulty to determine whether the pupil was shifted to the left or right within the eye region under near-threshold presentation duration. Regarding the context effect, we expected to observe the identical ordinal result pattern as in Experiments 1 and 2 for accuracy rates, RTs, and confidence ratings, independent of the third response option.

We conducted two experiments (Experiments 3a and 3b). In Experiment 3a, we presented each target (direct vs. left vs. right gaze) in one third of the trials in order to ensure that the ratio of target presentation was equal across all three gaze conditions (cf. Experiment 2a). Consequently, the stimulus ratio was 1:2 regarding the ratio of direct versus averted gaze targets. To rule out that the more frequent presentation of averted gaze targets modulates the effect, direct gaze targets were presented in half of the trials, while left and right gaze targets were presented at equal ratio in the remaining half of the trials in Experiment 3b.

The psychophysical approach of SDT has been developed to quantify the influence of the two factors sensitivity and response bias on performance in tasks where two different types of stimuli must be discriminated (Stanislaw & Todorov, 1999). The experimental design of Experiment 3 involved the discrimination between three response alternatives (direct vs. left vs. right gaze). To overcome this, we computed individual *d’* measures by conducting separate standard SDT analyses for each gaze direction and stimulus condition where one of the gaze directions was always treated as signal, while the other two served as noise^2^. Since this is not a standard procedure for which SDT analyses have originally been developed, and since values are not perfectly comparable to those of Experiments 1 and 2, we refrained from analyzing *d’* and *c* values in Experiment 3 and focused on the analysis of accuracy rates and RTs instead. We only relied on *d’* values as a potential exclusion criterion, as in the previous experiments.

### Experiment 3A

#### Method

##### Participants

We recruited 24 new participants. The data of three participants had to be excluded (individual *d*s < 0). The data of the remaining 21 participants were analyzed (mean age: 25.1 years, *SD* = 4.6 years, age range: 19–36 years, four male). All participants received monetary compensation for participation, reported normal or corrected-to-normal vision and provided written informed consent before participation. On average, the perceived task difficulty amounted to 4.7 points (*SD* = 1.9) on the same 9-point scale as used in the previous experiments.

##### Apparatus, stimuli, and procedure

In Experiment 3a, the identical technical equipment and stimulus material as in Experiment 1 was used. The procedure was similar to Experiment 1, but we implemented some changes. Unlike in the previous experiments, participants had to decide between three response options, that is, “left” versus “direct” versus “right” gaze. Participants responded via keypress (left/direct/right mapped onto numbers 1-3 of the numeric keypad). To avoid confusion due to a counterintuitive assignment of response options to response keys, we refrained from balancing the assignment of response options to response keys. Thus, the response option “left” was always mapped to number “1” (and presented on the left side of the screen) and the response option “right” to number “3” (and presented on the right side of the screen), while response option “direct” was mapped to number “2” (and presented centrally). Stimuli were presented in a fully randomized order in nine blocks of 56 trials each (resulting in 504 trials in total) with breaks in between blocks. Each stimulus and gaze condition appeared equally often. We additionally informed participants at the beginning of the experiment (via screen-based instructions) that each gaze condition would be presented equally often to avoid any keypress biases toward the central key, especially in case of high uncertainty regarding the correct answer. This information was repeated after/prior to each block.

##### Design and analyses

We extended the analyses to also include the third response option by performing 3 × 3 repeated-measures ANOVAs with the factor context (upright vs. inverted vs. eyes) and gaze (left vs. direct vs. right) as within-subjects factors for accuracy rates, confidence levels, and RTs. Like in the previous experiments, we removed all incorrect responses prior to analyzing RTs (16.70% of the data). Then, 0.40% of the data were eliminated due to anticipatory responses (RTs < 100 ms) or extremely high RTs (RTs > 5,000 ms). Additionally, we removed 3.60% of the data due to outlier correction (RTs > 2.5 SDs from the corresponding cell mean; calculated separately per participant and condition).

#### Results

##### Accuracy

The repeated-measures ANOVA revealed an effect of context, *F*(2,40) = 30.77, *p* < .001, *ƞ*^2^_p_ = .61 (see Fig. 3), with highest accuracy for upright faces, lower accuracy for inverted faces, and lowest accuracy for the eyes-only condition (see Table 2). Paired *t*-tests indicated significant differences between all context conditions (*p*s < .05). The effect of gaze was significant, *F*(2,40) = 5.71, *p* = .007, *ƞ*^2^_p_ = .22, with reduced accuracy for direct gaze (*M* = 78.88%, *SE* = 3.07%) compared to both left (*M* = 86.76%, *SE* = 2.60%), *t*(20) = 3.67, *p* = .002, *d* = 0.80, and right gaze (*M* = 84.27%, *SE* = 2.91%), *t*(20) = 1.84, *p* = .080, *d* = 0.40. Left and right gaze did not significantly differ, *t*(20) = 1.27, *p* = .220, *d* = 0.28. The interaction was significant, *F*(4,80) = 3.39, *ε* = .47, *p* = .047, *ƞ*^2^_p_ = .15. Separate one-way ANOVAs for each context condition were conducted to specify the interaction. While there was no effect of gaze on accuracy for inverted faces, *F*(2,40) = 0.42, *p* = .657, *ƞ*^2^_p_ = .02, we observed a significant gaze effect for both upright faces, *F*(2,40) = 5.90, *p* = .006, *ƞ*^2^_p_ = .23, and the eyes-only condition, *F*(2,40) = 6.89, *ε* = .72, *p* = .007, *ƞ*^2^_p_ = .26. For upright faces, the effect of gaze was driven by higher gaze accuracy rates for left gaze compared to both right gaze, *t*(20) = 3.43, *p* = .003, *d* = 0.75, and direct gaze, *t*(20) = 3.31, *p* = .003, *d* = 0.72, while direct and right gaze conditions did not significantly differ, *t*(20) = 0.05, *p* = .965, *d* = 0.01. In the eyes-only condition, the effect of gaze was due to reduced accuracy rates for direct gaze compared to both left gaze, *t*(20) = 3.26, *p* = .004, *d* = 0.71, and right gaze, *t*(20) = 2.17, *p* = .043, *d* = 0.47, while left- and right-gaze conditions did not differ, *t*(20) = 1.71, *p* = .104, *d* = 0.26. Analyzing only high-confidence trials revealed a very similar pattern of results, as demonstrated by the significant effect of context, *F*(2,40) = 22.90, *p* < .001, *ƞ*^2^_p_ = .53, the significant effect of gaze, *F*(2,40) = 16.86, *ε* = .67, *p* < .001, *ƞ*^2^_p_ = .46, and the significant interaction, *F*(4,80) = 10.14, *ε* = .44, *p* = .001, *ƞ*^2^_p_ = .34.

##### Response times

The repeated-measures ANOVA showed a significant main effect of context, *F*(2,40) = 20.49, *p* < .001, *ƞ*^2^_p_ = .51 (see Fig. 4), revealing the typical pattern: On average, participants responded fastest in the upright condition, slower in the inverted condition, and slowest in the eyes-only condition (see Table 2). Pairwise *t*-tests showed significant pairwise differences between all context conditions (*p*s < .001). We observed fastest RTs for the right-gaze condition (*M* = 516 ms, *SE* = 47 ms), slower RTs for the left-gaze condition (*M* = 558 ms, *SE* = 46 ms), and slowest RTs for the direct gaze condition (*M* = 659 ms, *SE* = 51 ms), *F*(2,40) = 24.58, *p* < .001, *ƞ*^2^_p_ = .55. Pairwise *t*-tests indicated significant differences between all gaze conditions (*p*s < .05). The interaction was not statistically significant, *F*(4,80) = 2.27, *ε* = .53, *p* = .113, *ƞ*^2^_p_ = .10 .

##### Response confidence

Confidence ratings were significantly different across context conditions, *F*(2,40) = 52.64, *p* < .001, *ƞ*^2^_p_ = .73, reflecting the pattern as observed for accuracy and RTs: Confidence ratings were highest in the upright condition (*M* = 4.10, *SE* = 0.15), lower in the inverted condition, (*M* = 3.59, *SE* = 0.17), and lowest in the in the eyes-only condition (*M* = 3.11, *SE* = 0.17). Paired *t*-tests indicated significant differences between all context conditions (*p*s < .001). There was a significant effect of gaze, *F*(2,40) = 26.13, *p* < .001, *ƞ*^2^_p_ = .57. Participants reported reduced confidence ratings for direct gaze (*M* = 3.37, *SE* = 0.16) compared to both left gaze (*M* = 3.73, *SE* = 0.15) and right gaze (*M* = 3.70, *SE* = 0.16), both *p*s < .001. The confidence ratings for left and right gaze did not differ significantly, *t*(20) = 0.69, *p* = .495, *d* = 0.15. The interaction was significant, *F*(4,80) = 2.90, *p* = .027, *ƞ*^2^_p_ = .13. Separate one-way ANOVAs per each context condition demonstrated that the interaction was driven by a larger gaze effect in the inverted and eyes-only condition, *F*(2,40) = 13.37, *p* < .001, *ƞ*^2^_p_ = .40, and *F*(2,40) = 20.81, *p* < .001, *ƞ*^2^_p_ = .51, respectively, as compared to the upright condition, *F*(2,40) = 6.06, *ε* = .78, *p* = .010, *ƞ*^2^_p_ = .23.

### Experiment 3B

#### Method

##### Participants

We recruited 24 new participants. The data of one participant had to be excluded because this participant had *d’* values lower than zero in at least one condition. The data of the remaining 23 participants were analyzed (mean age: 23.8 years, *SD* = 3.5 years, age range: 19–34 years, four male). Participants reported normal or corrected-to-normal vision, were paid for participation and gave written informed consent before the experiment started. On average, the perceived task difficulty amounted to 4.5 points (*SD* = 2.0) on the same 9-point scale as used in the previous experiments.

##### Apparatus, stimuli, and procedure

Experiment 3b was identical to Experiment 3a except for some minor changes regarding the relative frequency of stimulus presentation and the number of trials. The equal ratio of direct versus left versus right gaze as implemented in Experiment 3a was changed to a ratio of 2:1:1 (direct vs. left vs. right gaze) in order to present direct and averted gaze in 50% of trials each. As in Experiment 3a, we informed participants at the beginning of the experiment, as well as prior to each block, about this frequency distribution via on-screen instructions. Trials were presented in ten blocks of 48 trials each (resulting in a total of 480 trials) with breaks in between blocks.

##### Design and analyses

We conducted the same analyses as reported for Experiment 3a. Prior to analyzing RTs, we removed all incorrect responses (19.05% of the data). After that, we eliminated 0.75% of the data due to anticipatory responses (RTs < 100 ms) or extremely high RTs (RTs > 5,000 ms). Last, we removed 3.34% of the data due to outlier correction (RTs > 2.5 SDs from the corresponding cell mean; calculated separately per participant and condition).

#### Results

##### Accuracy

Again, accuracy was highest for upright faces, lower for inverted faces, and lowest for the eyes-only condition (see Table 2), yielding a significant effect of context, *F*(2,44) = 30.41, *p* < .001, *ƞ*^2^_p_ = .58 (see Fig. 3). Paired *t*-tests indicated significant differences between all context conditions (*p*s < .05). There was no significant effect of gaze, *F*(2,44) = 2.03, *ε* = .64, *p* = .163, *ƞ*^2^_p_ = .08, and also the interaction was not significant, *F*(4,88) = 2.45, *ε* = .56, *p* = .091, *ƞ*^2^_p_ = .10. Based on the result pattern as observed for accuracy rates in Experiment 3a, we conducted the following explorative analysis: Separate one-way ANOVAs for each context condition revealed an effect of gaze for upright faces, *F*(2,44) = 5.04, *p* = .011, *ƞ*^2^_p_ = .19. Accuracy rates were highest for left gaze (91.30%, *SE* = 2.02%), and significantly higher for left gaze compared to both direct gaze (85.65%, *SE* = 2.36%), *t*(22) = 2.60, *p* = .016, *d* = 0.54, and right gaze (87.39%, *SE* = 2.11%), *t*(22) = 2.74, *p* = .012, *d* = 0.57. The difference between direct and right gaze was not significant, *t*(22) = 0.97, *p* = .341, *d* = 0.20. There was neither a significant effect of gaze for inverted faces, *F*(2,44) = 2.45, *ε* = .68, *p* = .120, *ƞ*^2^_p_ = .10, nor for the eyes-only condition, *F*(2,44) = 1.79, *ε* = .66, *p* = .192, *ƞ*^2^_p_ = .08. The analysis of high-confidence trials showed a very similar pattern of results, as demonstrated by the significant effect of context, *F*(2,44) = 19.83, *ε* = .77, *p* < .001, *ƞ*^2^_p_ = .47, but no significant effects concerning gaze, *F*(2,44) = 3.82, *ε* = .61, *p* = .054, *ƞ*^2^_p_ = .15, or the interaction, *F*(4,88) = 1.56, *ε* = .48, *p* = .222, *ƞ*^2^_p_ = .07.

##### Response times

Responses were fastest in the upright condition, slower in the inverted condition, and slowest in the eyes-only condition, yielding a significant context effect, *F*(2,44) = 15.66, *ε* = .76, *p* < .001, *ƞ*^2^_p_ = .42 (see Fig. 4 and Table 2). Pairwise *t*-tests indicated significant differences for the upright condition compared to both the inverted and the eyes-only condition (*p*s < .001), while the inverted and the eyes-only condition did not significantly differ, *t*(22) = 0.80, *p* = .434, *d* = 0.17. Further, participants responded fastest in the right gaze condition (*M* = 537 ms, *SE* = 29 ms), at an intermediate level in the left-gaze condition (*M* = 598 ms, *SE* = 29 ms), and slowest in the direct gaze condition (*M* = 656 ms, *SE* = 35 ms), *F*(2,44) = 15.29, *p* < .001, *ƞ*^2^_p_ = .41. Pairwise *t*-tests indicated significant differences between all gaze conditions (*p*s < .05). The interaction was not statistically significant, *F*(4,88) = 2.00, *p* = .101, *ƞ*^2^_p_ = .08.

##### Response confidence

Participants indicated highest confidence for upright faces (*M* = 3.91, *SE* = 0.14), lower confidence for inverted faces (*M* = 3.54, *SE* = 0.15), and lowest confidence for the eyes-only condition (*M* = 3.16, *SE* = 0.16), revealing a significant effect of context *F*(2,44) = 41.05, *p* < .001, *ƞ*^2^_p_ = .65. Every pairwise *t-*test between context conditions was significant (*p*s < .001). The effect of gaze was significant, too, *F*(2,44) = 10.10, *ε* = .63, *p* = .002, *ƞ*^2^_p_ = .32. Participants were less confident in the direct condition (*M* = 3.34, *SE* = 0.12) compared to both left condition (*M* = 3.64, *SE* = 0.16), *t*(22) = 3.31, *p* = .003, *d* = 0.69, and right condition (*M* = 3.63, *SE* = 0.16), *t*(22) = 3.31, *p =* .003, *d* = 0.69. For left and right conditions, confidence ratings were not significantly different, *t*(22) = 0.30, *p* = .770, *d* = 0.06. The interaction was significant, *F*(4,88) = 2.88, *p* = .027, *ƞ*^2^_p_ = .12, indicating that the loss in confidence for direct as compared to left and right gaze was most pronounced in the eyes-only condition.

### Experiment 3: Discussion

Data of Experiment 3 are well in agreement with what we have observed in Experiments 1 and 2: Both accuracy rates and RTs were affected by the facial context showing the identical ordinal result pattern as reported in the previous experiments. The focus of Experiment 3 was to test whether the averted-gaze advantage (in terms of faster RTs and higher accuracy for averted vs. direct gaze direction) as observed in Experiments 1 and 2 is robust against variations in task demands. To do so, the task of Experiment 3 included not only distinguishing between direct and averted gaze, but also between direct, left, and right gaze. We reasoned that the performance advantage for averted gaze could disappear, or even reverse, because of the higher visual requirements when the direction of an averted gaze stimulus needs to be identified under conditions of near-threshold presentation. The results were straightforward: There was no indication for a direct-gaze advantage in neither accuracy nor RTs. On the contrary, and similar to Experiments 1 and 2, performance was considerably faster for targets displaying averted gaze (to the left or right) as compared to targets displaying direct gaze.

The analysis of accuracy rates revealed somewhat less clear-cut results, since an effect of gaze was not present across all context conditions: In Experiment 3a, we observed a clear averted-gaze advantage for the upright-face and eyes-only condition, while there was no significant averted-gaze advantage when faces were presented upside-down. In Experiment 3b, explorative analyses revealed a tendency toward the absence of significant gaze effects for both the eyes-only and the inverted-face condition. For upright faces, results showed a left-gaze advantage in Experiment 3a and a tendency toward a left-gaze advantage in Experiment 3b. This inconsistency across experiments regarding the interaction of face context and gaze will be further covered in the *General discussion*.

This left-gaze advantage was only present when faces were presented in upright position: Accuracy rates were highest for left gaze and reduced, but on a comparable level, for both direct and right gaze. At first sight, one could speculate that the left-visual-field-advantage (LVF-advantage) advantage for perception of gaze direction (Palanica & Itier, 2011; Ricciardelli, Ro, & Driver, 2002) might drive this left-gaze advantage. The LVF-advantage proposes that judgments about gaze directions are more affected by the eye presented in the LVF as compared to the eye presented in the right visual field (RVF), and is consistent with findings from neuroscience showing a right-hemispheric dominance in gaze processing (e.g., Sato, Kochiyama, Uono, & Toichi, 2016; Wicker, Michel, Henaff, & Decety, 1998). However, in the present study, the critical gaze direction information, that is, whether the pupil was located centrally or at the left or right edge of the eye region was present in both the left and right eye of our target stimuli. Thus, the critical information was presented in both the LVF and the RVF within each trial, and the LVF-advantage for gaze perception should affect accuracy equally for both left and right gaze direction when stimuli are presented centrally. This may be different for peripheral presentations of gaze targets (see below for a more detailed discussion on stimulus position).

## General discussion

The present series of experiments investigated performance in a gaze-discrimination task under conditions where direct vs. averted gaze stimuli in different facial contexts were briefly presented and masked. Participants were asked to indicate the perceived gaze direction as quickly and accurately as possible via keypress. Our results can be broken down into two key findings. First, the data show that accuracy, speed of judgment about gaze direction, and confidence ratings are influenced by the facial context in which the eyes are presented, demonstrating a *facial context effect*. Across all experiments, we observed an ordinal pattern with highest discrimination performance in terms of speed, accuracy and sensitivity for faces presented in upright position, lower performance for inverted faces, and lowest performance for eyes presented in isolation. The same pattern was evident for confidence ratings.

Second, our results demonstrate a clear *averted-gaze advantage* across all experiments in terms of faster RTs for averted versus direct gaze direction. The averted-gaze advantage was also present in accuracy rates with a slightly weaker effect in Experiment 3, where participants additionally had to discriminate between the two directions of the averted gaze targets (left or right). However, and in contrast to the averted-gaze advantage in RTs, the effect on accuracy was modulated by facial context in some experiments such that we observed the averted-gaze advantage for faces presented in upright position (across all experiments) and for eyes presented in isolation (in four out of five experiments), but no averted-gaze advantage for inverted faces in two experiments (Experiments 1 and 3). The averted-gaze advantage was also reflected in confidence ratings in four out of five experiments, demonstrating that participants also felt more confident in detecting averted (vs. direct) gaze. We discuss the two key findings and the following implications in more detail below, starting with the facial context effect.

### Effects of facial context

The effect of facial context, which we observed consistently across measures (accuracy, RTs, *d*’) and experiments, showed that gaze discrimination was severely disrupted by face inversion. This finding is typically referred to as *gaze inversion effect* (Schwaninger et al., 2005) and replicates previous results reporting a gaze inversion effect with respect to different behavioral measures including gaze discrimination ability (Jenkins & Langton, 2003) and gaze location judgments (Schwaninger et al., 2005). Converging evidence has been provided for the assumption that the gaze inversion effect is mainly caused by the orientation-sensitive processing of the eye region, rather than by the inversion of the facial context (Jenkins & Langton, 2003; Schwaninger et al., 2005; Senju & Hasegawa, 2006). Nevertheless, our data also suggest that removing the facial context, i.e., presenting the eye region in isolation, had detrimental effects on gaze discrimination, which was even beyond the effect of face inversion. Such a significant drop in discrimination performance has also been demonstrated by Jenkins and Langton (2003) and Langton et al. (2004), and suggests that the processing of the face as a whole contributes to the recognition of gaze direction. Briefly summarized, these authors argued that removing facial context information goes along with a loss of information regarding head orientation, which itself significantly contributes to the perception of gaze direction (Jenkins & Langton, 2003; Langton et al., 2004). Thus, it appears that gaze discrimination is not merely an outcome of focusing attention on task-relevant stimulus regions (the eyes), but rather a more complex process involving the integration of local (eyes) and global (face) outcomes of attentional processing. Note that not all previous studies on this issue reported evidence for the usefulness of facial context. For example, Schwaninger et al. (2005) reported comparable gaze location judgments irrespective of whether facial context information was provided or not. Note, however, that their task (Schwaninger et al., 2005) differed from our present task in several aspects, for example with respect to target presentation duration (short versus continuous presentation of the target) or response format (perceived gaze direction vs. perceived gaze location judgments), which might explain the diverging results.

The poor performance observed in the eyes-only condition might also be traced back to factors other than the absence of facial context information as described above, for instance, to perceptual contrast phenomena. Unlike in the upright/inverted stimulus condition, where the eye region was implemented within the face, the eyes in the eyes-only condition were rather immediately surrounded by a high-contrast contour between the skin color and the black background (see Fig. 1 for a depiction of the stimulus material). This high contrast may have impeded a processing of the information within the eye region, especially given the short image presentation duration used in the current study. Future studies should explicitly address such perceptual contrast phenomena.

In this context, also the general question of whether the facial context effect as observed in the present series of experiments is specific to conditions of extremely brief target presentation (as studied here) or could also generalize to conditions where targets are presented for a longer duration should be considered in future studies. If it were rather the stimulus material (upright/inverted/eyes only) that drives the observed facial context effect, we would expect to obtain the same pattern of results even at longer presentation duration.

Another possibility of explaining some aspects of the context effect is by taking the nature of the masks into account. Masks were created such that they covered the area where the stimulus was previously presented. Hence, they were larger for upright/inverted face stimuli as compared to eyes only stimuli, where the mask only covered the eye region. This was done to ensure that the mask covers exactly the region of the stimulus in all conditions. As this design decision implies different mask sizes in these particular conditions, this might theoretically have affected the effectiveness of the masks. Note, however, that our pattern of results is generally in line with previous reports of low performance in eyes-only conditions (Jenkins & Langton, 2003; Langton et al., 2004). Additionally, it is important to keep in mind that the specific comparison of the eyes-only condition with the two face conditions was not the main focus of the present study, and it is beyond the scope of this paper to finally resolve potential effects of mask size on task performance.

### Averted-gaze advantage

Participants responded faster and more accurately to averted gaze stimuli in our gaze-discrimination task. Even in the few contexts where we did not observe a clear averted-gaze advantage, our data provide no indication whatsoever of a direct-gaze advantage. These results are especially interesting since they appear to be in contrast to the repeatedly reported superiority of direct over averted gaze across different context settings and task demands (Böckler et al., 2014; Palanica & Itier, 2012; Senju & Hasegawa, 2005; Stein et al., 2011). However, a closer look reveals that these previous studies differ from our present study in some important aspects that appear crucial for observing either a direct- or averted-gaze advantage. While former studies investigated the *detection* of constantly presented (gaze) targets from the surrounding peripheral environment (or the viewing time for computer agents with direct and averted gaze as in the study of Palanica and Itier, 2012), our study focused on the ability to *discriminate* gaze directions in stimuli that were briefly presented centrally. On a more applied level, comparing these studies would be equivalent to the comparison of the behavior of a person passing through a crowded pedestrian zone trying to find somebody as quickly as possible with the behavior of a person watching TV and trying to determine whether the gaze of a briefly displayed person was directed towards the viewer or not. Thus, we suggest that the averted-gaze advantage of the present study is mediated by mechanisms other than those involved in the “stare-in-the-crowd” (von Grünau & Anston, 1995; Senju et al., 2005) or “attention-capture-by-direct-gaze” effects (Böckler et al., 2014; Boyer & Wang, 2018; Palanica & Itier, 2012). For example, it could be of great importance whether gaze is perceived in the periphery (to capture our attention) or at the fovea (to extract information from the other’s gaze regarding new locations of interest). Nevertheless, given the convincing effect sizes and the replicability of the present averted-gaze advantage, we conclude that direct gaze is not generally associated with processing advantages irrespective of situational and task demands.

One possible way to explain the averted-gaze advantage could be to assume that participants regularly had trouble in judging gaze directions and resorted to a strategy involving a response bias in terms of faster and more frequently responding with “averted gaze,” especially in the eyes-only condition where the averted-gaze advantage was particularly pronounced. Such a pattern of results would be consistent with the results of a previous study reporting more *averted* responses in a gaze categorization task when only eyes were presented compared to when an upright face was visible (Mareschal et al., 2013). At first sight, this explanation of the averted-gaze advantage appears to be backed up by our data indicating a small response bias towards responding with “averted gaze.” Although not statistically significant, the absolute values of the response criterion *c* in our Experiments 1 and 2 were highest for the eyes-only condition mirroring the results reported by Mareschal et al. (2013). However, several other observations prevent us from explaining our results solely with respect to a response bias. First, accuracy was relatively high, indicating that participants did not generally experience difficulties in judging gaze direction. Additionally, participants indicated that they felt more confident when judging averted (vs. direct) gaze, suggesting that they indeed experienced greater ease when processing averted gaze. It is worth noting that we found the averted gaze effect also for "confident" trials, and not only in "unconfident" trials, suggesting that the effect was not merely driven by a corresponding response bias under high uncertainty. Taken together, it appears rather unlikely that the present averted-gaze advantage in RTs and confidence ratings can solely be explained as resulting from a response bias under uncertainty. Rather, it seems to represent a genuine processing advantage for averted gaze. One way to directly address this issue more explicitly in future studies would be to compare *d’* measures for direct and averted gaze when each gaze type is combined with a comparable reference (e.g., closed important set of results is related to the dependency of the averted-gaze advantage on facial context. First, consideration is given to the comparison of upright and inverted faces. In *accuracy rates*, the averted-gaze advantage was somewhat less stable when faces were presented upside-down (see Experiment 3, where we did not observe an averted-gaze advantage for inverted faces and Experiment 1, where we observed a corresponding trend). A similar observation was already reported in visual search paradigms (Senju et al., 2005), albeit with respect to the modulation of a *direct-*gaze advantage dependent upon face orientation (upright versus inverted). Specifically, Senju and colleagues observed an elimination of the direct-gaze advantage for inverted faces. In contrast, the averted-gaze advantage in our study was present independent of face inversion in the majority of experiments in *RTs* (see Experiment 2a for an exception, where the averted-gaze advantage occurred only in the eyes-only condition). Similarly, Stein et al. (2011) and Chen and Yeh (2012) reported gaze effects in RTs that were independent of face inversion. They found that the representation of direct gaze is enhanced compared to averted gaze independent of face inversion even when gaze was processed unconsciously. Thus, the presence or absence of gaze effects dependent upon face orientation as reported by Senju et al. (2005), Stein et al. (2011), and Chen and Yeh (2012) might be due to conscious as opposed to unconscious processing of the gaze targets. With respect to the present results, one could suggest that accuracy rates rather represent a more conscious measure of gaze processing given that we observed inversion effects on accuracy rates (cf. Senju et al., 2005), while RTs rather resemble a more unconscious measure of gaze processing given that we did not observe inversion effects in RTs (cf. Chen & Yeh, 2012; Stein et al., 2011). However, this is clearly a speculative post hoc interpretation of our results, and one should always bear in mind that Senju et al. (2005), Stein et al. (2011), and Chen and Yeh (2012) observed a direct- (instead of averted-) gaze advantage in the first place.

A further relevant finding comes from a closer inspection of the eyes-only condition, which turned out to be the condition where the averted-gaze advantage was strongest and most reliably observed in both accuracy rates and RTs (except for Experiment 3b, where the averted-gaze advantage was less consistently observed given that in the eyes-only condition, the averted-gaze advantage was only present in RTs, not in accuracy rates). This general observation is in line with what was already discussed with respect to Experiment 2 – namely that the averted-gaze advantage predominantly comes into play in conditions where gaze discrimination is most impaired. Thus, it seems that the recognition of direct (vs. averted) gaze suffers more strongly from the absence of any information on head orientation.

Interestingly, around the time of the present study, another (independent) research group reported first evidence for an averted-gaze advantage in a gaze-discrimination task (McCrackin & Itier, 2019). Their task somewhat differed from the present task set, for example with respect to target presentation duration (500 ms vs. 35 ms in the present study) and the use of masks (blank screen vs. scrambled faces in the present study). The results of McCrackin and Itier (2019) are important with respect to both the replicability and the generalization of the present results: The averted-gaze advantage as observed in a gaze-discrimination task does not appear to be restricted to both the present stimulus set and the brief presentation duration of the gaze targets, but instead generalizes to other gaze-discrimination tasks.

While the majority of previous studies showed processing advantages for direct versus averted gaze, our present study reliably demonstrated evidence for an averted-gaze advantage in terms of faster RTs and higher accuracy for averted (vs. direct gaze) in a gaze-discrimination task. To delineate the underlying mechanisms of this effect, it is necessary to systematically address the differences in study design, such as stimulus position, presentation duration, and task set (detection vs. discrimination, etc.). In particular, stimulus position (central vs. peripheral) might be an important factor. It is known from different studies that foveal and peripheral vision differs quantitatively, and that specific types of stimulus changes, such as orientation changes, are difficult to discriminate peripherally (To, Gilchrist, Troscianko, & Tolhurst, 2011). In the context of gaze processing, it has been demonstrated that both head orientation and degree of eccentricity affect gaze perception (Florey, Clifford, Dakin, & Mareschal, 2015). Recently, it has also been shown that the effect of attention capture by direct gaze and dynamic motion on social attention is restricted to conditions where participants were instructed to maintain fixation at the center of the screen (Boyer & Wang, 2018). Moreover, the role of foveal versus peripheral vision has been explicitly addressed with respect to their respective contribution to visual search (Hughes, Southwell, Gilchrist, & Tolhurst, 2016), indicating the importance of peripheral information in visual search tasks. In total, these examples from various areas of research suggest that central versus peripheral presentation of the gaze target might be an important factor to consider. Future studies should explicitly address this issue. While a systematic manipulation of these factors is clearly beyond the scope of the present study, our set of results allows us to rule out some potentially confounding factors: First, we can rule out that the stimulus ratio influenced the averted-gaze advantage, since we did not observe any qualitative differences in performance pattern between experiments with different stimulus ratio. Second, the averted-gaze advantage is not moderated by task demands such as whether discrimination between direct and averted gaze direction only, or additionally between directions of the averted gaze to the left and right is required.

### Response confidence

We included a measure of response confidence after each trial to compare the objective performance measures with the participants’ subjective perception of their discrimination performance. The analysis of these confidence ratings revealed straightforward results: Consistently across all experiments, confidence was highest for upright faces and decreased for both inverted faces and eyes presented in isolation. Additionally, confidence was higher for averted (vs. direct) gaze direction (except for Experiment 2b where the gaze effect just barely missed significance). Thus, confidence mirrored our accuracy-related results quite consistently across experiments, similar to previous observations by Anderson et al. (2016). As an exception to this general pattern, confidence ratings and accuracy rates were dissociated under certain conditions in Experiments 1 and 3 such that the averted-gaze advantage was present or absent depending on face context in accuracy rates, while it was robustly reflected in confidence ratings independent of facial context. Gaze effects were predominantly pronounced in accuracy rates when faces were presented upside-down or when eyes were presented in isolation (Experiment 1 and Experiment 3a) or only present when eyes were presented in isolation (Experiment 3b), as opposed to higher confidence ratings for averted versus direct gaze targets independent of facial context. This dissociation is interesting with respect to the notion that direct gaze receives privileged processing outside of conscious awareness (Stein et al., 2011). It suggests that the perception of direct gaze is not necessarily bound to the conscious awareness of the gaze direction, especially under conditions where the perception of gaze discrimination is generally impaired (as indexed by the drop in accuracy rate for inverted faces and eyes presented in isolation in the present study).

### Concluding remarks

To conclude, the present series of experiments demonstrated an *averted-gaze advantage*, which has shown to be robust and replicable across different measures (RT, accuracy, and confidence measures) and experimental settings in the present gaze-discrimination task. This finding appears to have important implications for the study of gaze processing when it comes to determine under which situational and task demands direct gaze receives privileged processing over averted gaze, or vice versa. Though preliminary, it seems plausible to assume that effective processing and detection of averted gaze is important when this averted gaze is presented briefly at the current fixation location in order to establish intersubjective attention alignment (i.e., gaze cueing) in a highly efficient manner. In contrast, when direct gaze is presented in the periphery, it might be of social relevance to detect these communicative approach signals efficiently.

## Footnotes

1 Note that “gaze” could not be included as a factor here due to the inherent logic of the SDT analysis: The sensitivity parameter *d*’ measures the ability to distinguish signals (here: direct gaze targets) from noise (here: averted gaze targets) in different facial contexts. Consequently, the sensitivity parameter *d*’ combines accuracy scores for direct and averted gaze targets within a single measure.

2 This method resulted in nine separate *d’* values per participant (one *d’* value for each gaze direction of each stimulus condition). For example, for *d’*_direct gaze_, the corresponding hit rate was computed as the proportion of “direct gaze” responses when direct gaze was presented, and the corresponding false alarm rate was computed as the proportion of “direct responses” in the remaining noise trials (that is, left or right gaze). Doing so implies that there are ambiguous trials: If participants incorrectly respond to a left-gaze stimulus with a keypress indicating right gaze, these trials will be counted as correct rejections in terms of SDT, since noise has correctly been identified as noise. As a consequence, the sensitivity to discriminate gaze direction is overestimated (due to inflated correct rejection rates, which lead to rather low false alarm rates, eventually causing high d’ values). To correct for sensitivity overestimation, we excluded these ambiguous trials for the calculation of *d‘*.
